# The Clinical Features of Infantile Hypertrophic Pyloric Stenosis in Chinese Han Population: Analysis from 1998 to 2010

**DOI:** 10.1371/journal.pone.0088925

**Published:** 2014-02-19

**Authors:** Zhiqiang Feng, Yuqiang Nie, Youxiang Zhang, Qingning Li, HuiMing Xia, SiTang Gong, Hai Huang

**Affiliations:** 1 Department of Gastroenterology, Guangzhou Digestive Disease Center, Guangzhou First People's Hospital, Guangzhou Medical University, Guangzhou, China; 2 Guangzhou Key Laboratory of Digestive Disease, Guangzhou First People's Hospital, Guangzhou Medical University, Guangzhou, China; 3 Department of Pediatrics, Guangzhou First People's Hospital, Guangzhou Medical University, Guangzhou, China; 4 Department of Surgery, Guangzhou Women and Children's Medical Center, Guangzhou, China; 5 Department of Gastroenterology, Guangzhou Women and Children's Medical Center, Guangzhou, China; National Taiwan University Hospital, Taiwan

## Abstract

**Objective:**

To investigate clinical features of infantile hypertrophic pyloric stenosis (IHPS) in Chinese Han population.

**Methods:**

Three hundred and sixteen hospitalized patients with IHPS from January 1998 to February 2010 were retrospectively reviewed, and data including patient's sex, onset age, other coexisting congenital defects, pyloric circular muscle thickness evaluated by ultrasonograph, serum electrolytes concentration, and results of arterial blood gas analysis on admission were collected. The patients were divided into two groups: the duration between first onset and admission less than or equal to 10 days (early onset group), and more than 10 days (late onset group). The results of arterial blood gas and serum electrolyte concentration were compared between the two groups.

**Results:**

There were 271 males and 45 females in 316 patients; the onset age ranged between 1 and 351 (26.5±26.6) days. The birth weight ranged between 1.6 and 4.5 (3.23±0.44) kilograms; coexisting congenital defects were found in 65 cases (20.6%). Pyloric circular muscle thickness was 4–8 (5.4±1.0) millimetres (mm). For the early onset group, the rates of hypokalemia, hypochloraemia and hypercapnia were significantly lower than those in the late onset group (18.67% VS 50%, *P*<0.0001; 46.03% VS 71.01%, *P* = 0.003; 56.58% VS 83.44%, *P* = 2.17×10^−5^; respectively).

**Conclusions:**

The symptom duration in Chinese Han population was longer than that in other populations. And as the prolongation of symptom duration, the incidence of acid-base imbalance increased significantly. Infants with persistent vomiting at the age of 3∼5 weeks after birth should be considered IHPS, and go to hospital as soon as possible in order to reduce the incidence of hypokalemia, hypochloraemia and hypercapnia, and avoid deterioration.

## Introduction

Infantile hypertrophic pyloric stenosis (IHPS) is a condition affecting young infants, in which gastric outlet obstruction results from hypertrophy and thickening of the circular muscle of the pylorus. It has been shown that IHPS occurs much more frequently in males than in females [1], [2], [3], [4], [5], [6]. The incidence rate in Caucasian is higher than that in Afro-Caribbeans and Asians [2], [7], [8], [9], [10]. Its etiology is still unclear, undoubtedly includes environmental and genetic components [11], [12]. The typical clinical presentation is projectile, non-bilious vomiting occurring at the age of 2∼8 weeks [2], [13], and usually occurring 10∼30 minutes after feeding. Although medical diagnosis can be made by palpable olive-shaped mass in the right upper quadrant, abdominal ultrasonography and barium studies are necessary in establishing the diagnosis [14].

To the best of our knowledge, there were no clinical feature analysis reports with IHPS in mainland China, although a lower incidence of IHPS in Chinese has been reported from American, Singapore and Taiwan [6], [9], [10]. Therefore, a retrospective investigation was conducted to describe the clinical features in Chinese infancy. A total of 316 patients hospitalized with IHPS have been reviewed. Data including sex, onset age, other coexisting congenital defects, pyloric circular muscle thickness, serum electrolytes concentration and arterial blood gas were collected.

## Patients and Methods

### 1. Patients and methods

The study was approved by the Ethics Committee of Guangzhou First People's Hospital and Guangzhou Women and Children's Medical Center. Surgical informed consent was obtained from each infant's legal guardian before operation and was stored in the hospitals' Medical record room, undoubtedly before samples collection. From January, 1998 to February, 2010, all patients diagnosed with IHPS treated with open pyloromyotomy or laparoscopic pyloromyotomy, or endoscopic pyloromyotomy in both Guangzhou First People's Hospital and Guangzhou Women and Children's Medical Center were included in this study. From this retrospective series, all data was collected from medical records by two or three of the authors (Zhiqiang Feng, QingNing Li or Hai Huang). The diagnosis of IHPS was based on a history of projectile vomiting, palpation of an olive mass, and ultrasonography, upper gastrointestinal brium series and was confirmed with surgery.

The following data was retrospectively collected: demographics, clinical manifestations, birth order, birth weight, gestational age, other congenital malformations, ultrasonographic findings, serum electrolytes concentration and acid–base status. All these data gained before any therapeutic interventions. According to Huang [15], the timeframe of diagnosis was defined as the interval between age of symptom onset and age of diagnosis. All patients were divided into two groups based on the duration between first onset and admission, which is less than or equal to 10 days (early onset group), and more than 10 days (late onset group). The results of arterial blood gas and serum electrolyte concentration were compared between the two groups.

The data was expressed using frequencies, percentages or mean and standard deviation (SD). Chi-square tests were performed to analyze differences between the two groups. A *P* value<0.05 was defined as statistically significant. Statistical analysis was performed using SPSS 13.0 software package for windows (SPSS Inc, IL, USA).

## Results

### 1. General data of IHPS patients (Table 1)

**Table 1:** General data of IHPS.

Male to female ratio	271∶45
Birth weight (kilograms)	3.23±0.44(1.60∼4.50)
Symptom onset age(days)	26.5±26.6(1∼351)
Patterns of delivery	spontaneous delivery(204 cases)
	abdominal delivery(109 cases)
	ventouse-assisted vaginal delivery(3 cases)
Thickness of pyloric circular muscle(mm)	5.4±1.0(3.0∼8.0)
length of pyloric muscle (mm)	20.8±3.8(11.0∼38.0)
Birth order	firstborn child 76.3%(241/316)
	the second 19.6%(62/316)
Other congenital malformations	65 cases (20.6%)
Pre-term	13 cases (4.1%, 13/316).
Term	303 cases(95.9%)

**Figure 1:**
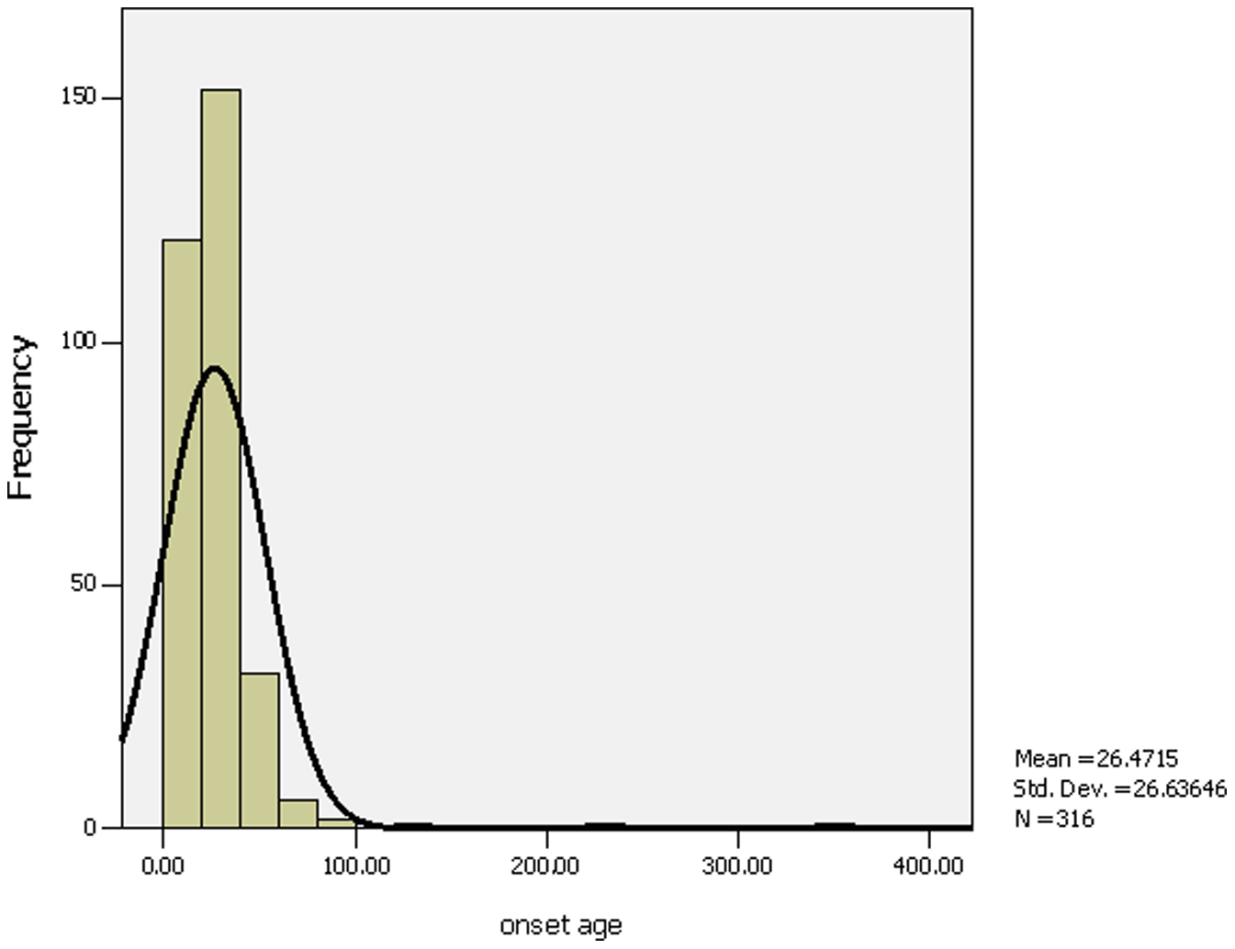
Age at onset (days). From the Figure 1, we found the age of symptom onset ranged between 1 and 351 days, and most onset age were 10 to 40 days.

There were 316 patients with IHPS during the past 12-year period. There were 271 boys and 45 girls. All patients had classic projectile,and persistent non-bilious vomiting. All the parents denied the familial genetic diseases and there were no use of macrolide antibiotics during pregnancy. The age of symptom onset ranged between 1 and 351 days, and the mean was 26.5±26.6 days (Figure 1). The duration from first onset to diagnosis was 24.9±25.1 days (range 1–273 days). One hundred and ninety six patients were full-term infants, and the gestational age of the remaining 120 cases ranged from 34 to 43 weeks (39.0±1.6 weeks). There were three patterns of delivery: spontaneous delivery (204 cases), abdominal delivery (109 cases) and ventouse-assisted vaginal delivery (3 cases).

The birth weight was 3.23±0.44 kilograms (ranging between 1.60 kilograms and 4.50 kilograms). The weight at presentation was 3.85±0.89 kilograms (range from 1.75 to 9.0 kilograms). The firstborn child was noted in 76.3% (241/316) of the cases, the second was 19.6% (62/316), 10 for third, two for forth and one for fifth, and a pair of twins affected. There were two triplet pedigrees, with one being affected in each pedigree. Premature birth was seen in 13 cases (4.1%, 13/316).Other coexisting other congenital malformations were found in 65 cases (20.6%). Fifty cases had one other congenital defect. Two other congenital defects were seen in 12 cases and three in 3 cases. The most common congenital defect concomitant with IHPS was congenital cardiovascular defects (50.8%, 33/65), followed by the gastrointestinal tract defects (24.6%, 16/65, Table 2) and central nervous system congenital defects (15.4%, 10/65).

**Table 2:** The details of coexisting congenital defect in gastrointestinal tract.

Congenital abnormalities	cases
Bilateral inguinal hernia	5
Right inguinal hernia	6
Left inguinal hernia	1
congenital malrotation of intestine	2
Umbilical hernia	1
esophageal hiatal hernia	1

### 2. The results of imaging findings

**Figure 2:**
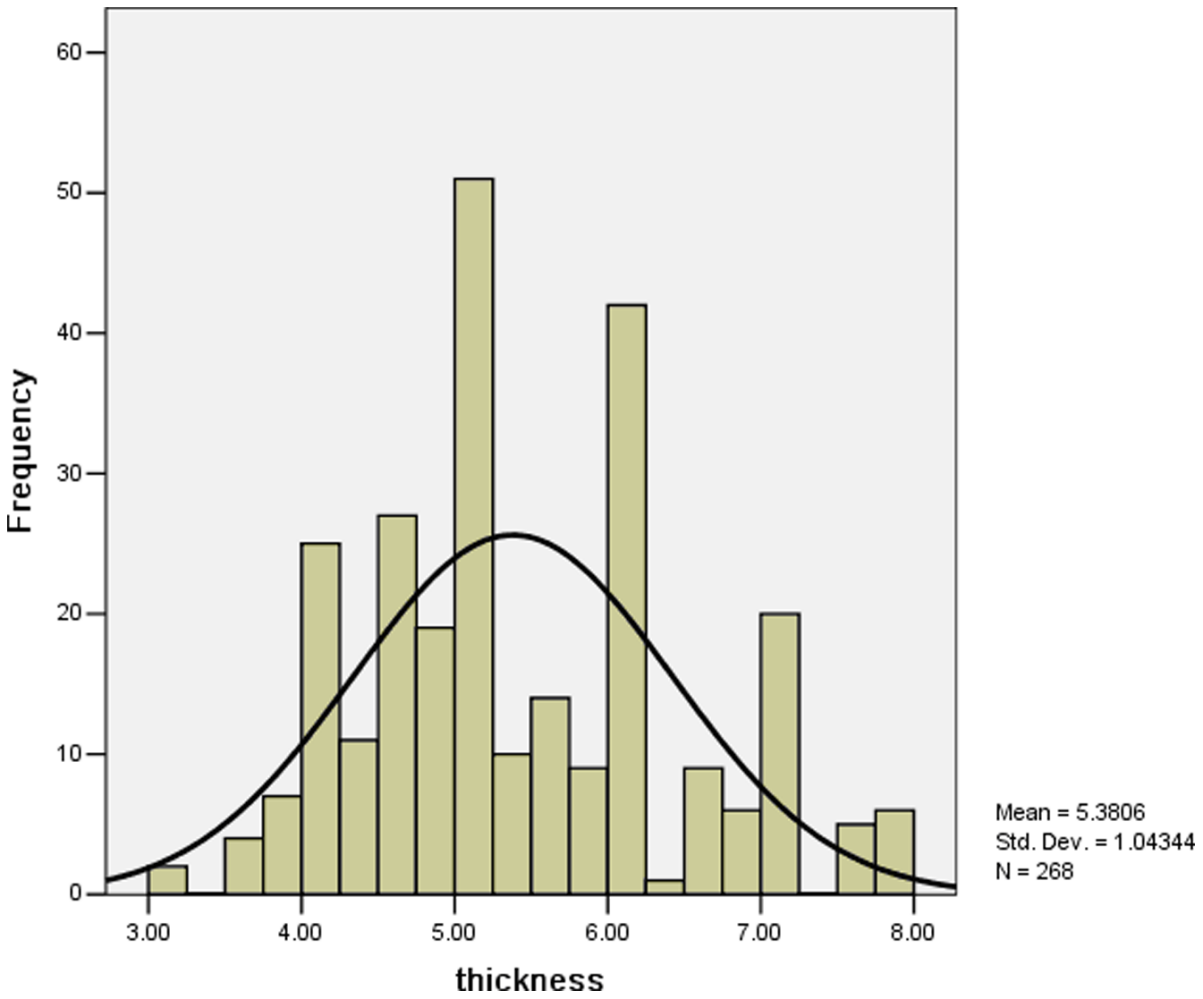
Thickness of pyloric circular muscle (mm). From the Figure 2, we found the mean thickness of pyloric circular muscle ranged from 3 to 8 mm.

In our two hospitals 273 cases adopting ultrasound examinations, in which 268 cases(268/273, 98.17%) were positive and 5 cases were negative which the diagnosis being confirmed by contrast barium (UGI) study. For 268 infants, the mean length of pyloric muscle was 20.8±3.8 mm (ranged 11.0∼38.0 mm), and the mean thickness of pyloric circular muscle ranged from 3 mm to 8 mm (5.4±1.0 mm, Figure 2). The remaining 43 infants had ultrasound examination or upper GI contrast study in other hospitals and lacked detailed records for both pyloric muscle thickness and pyloric muscle length.

### 3. The results of laboratory examination

The results of arterial blood gas analysis were available for 232 (73.42%) patients at admission. An elevated bicarbonate level (HCO_3_>25 mmol/L) was observed in 179 of 232 patients (77.16%) and pH was above 7.45 in 85.35% (198/232) of patients. Hypoxemia (PO_2_<10.7 kpa) was seen in 60.34% (140/232) of the patients. Serum sodium and potassium were available for 76.27% (241/316) and 75.63% (239/316) patients when admission, while serum chloride was available for 63.61% (201/316) patients. The hypochloraemia (Cl<95 mmol/L) was found in 127 of 201 measurements (63.18%). Hyponatraemia (<135 mmol/L) was seen in only 54.36% (131/242) of patients, with 40.17% (96/239) patients displaying hypokalemia (<3.5 mmol/L).

### 4. Comparison between early onset group and late group

All cases were divided into two groups according to the timeframe of diagnosis which was less than or equal to 10 days (early onset group), and more than 10 days (late onset group). There were 103 patients in early onset group, and 213 patients in late onset group.

Results of arterial blood gas analysis, serum electrolyte concentration, the Pyloric muscle thickness were shown in Table 3. There was no difference in birth weight, serum sodium concentration and PH between two groups, while there were significant differences in the mean pyloric muscle thickness (5.09±0.96 vs. 5.55±1.07 mm, *P* = 0.001), arterial partial pressure of oxygen (10.76±4.27 vs 8.86±4.24 kpa, *P* = 0.001), serum potassium concentration (4.07±0.65 vs 3.60±0.86, *P*<0.0001), bicarbonate level (26.75±6.66 vs 34.99±10.59, P<0.0001) and serum chlorine concentration (93.89±11.12 vs 86.69±12.78, P = 0.000158) between late and early onset groups (Table 3).The rates of hypokalemia, hypochloraemia and hypercapnia in the early onset group were significantly lower than those in the late onset group (Table 4).

**Table 3:** Comparison of some index between two groups.

	Early onset group(cases)	Late onset group(cases)	P value
Birth weight (kg)	3.19±0.47(97)	3.25±0.43(198)	0.233
length of pyloric muscle (cm)	2.00±0.37(89)	2.12±0.38(179)	0.026
thickness of pyloric muscle (mm)	5.08±0.95(89)	5.53±1.06(179)	0.001
PO_2_ (kpa)	10.76±4.27(76)	8.86±4.24(163)	0.001
PCO_2_ (kpa)	4.20±1.27(76)	5.20±1.54(163)	1.62×10^−6^
HCO_3_^−^ (mmol/L)	26.75±6.66(76)	34.99±10.59(163)	2.13×10^−9^
Serum chloride (mmol/L)	93.89±11.12(63)	86.69±12.78(138)	0.000158
Serum potassium (mmol/L)	4.07±0.65(75)	3.60±0.83(164)	2.34×10^−5^
serum sodium (mmol/L)	134.69±5.76(76)	133.06±6.27(165)	0.056
serum pH	7.54±0.10(76)	7.56±0.11(163)	0.182

Notes: detailed data not recorded in some medical records.

**Table 4:** Comparison of rates of examinations between two groups.

	Status	Early onset group(cases,%)	Late onset group(cases,%)	Pvalue
serumpotassium	Hypokalemia(<3.5 mmol/L)	14 (18.67%)	82 (50%)	<0.0001
	normal	61 (81.33%)	79 (48.17%)	
	Hyperkalemia(>5.5 mmol/L)	0	3 (1.83%)	
serumsodium	Hyponatraemia	37 (48.68%)	94 (56.97%)	0.198
	normal	39 (51.32%)	68 (41.21%)	
	hypernatremia	0	3 (1.82%)	
Serumchloride	Hypochloraemia	29 (46.03%)	98 (71.01%)	0.003
	normal	30 (47.62%)	36 (26.09%)	
	hyperchloraemia	4 (6.35%)	4 (2.90%)	
pH	>7.45	63 (82.89%)	135 (82.82%)	0.914
	normal	11 (14.48%)	25 (15.34%)	
	<7.35	2 (2.63%)	3 (1.84%)	
HCO_3_^−^	>25 mmol/L	43 (56.58%)	136 (83.44%)	2.17×10^−5^
	normal	33 (43.42%)	27 (16.56%)	
PO_2_	<10.7 kpa	38 (50%)	102 (62.58%)	0.066
	normal	38 (50%)	61 (37.42%)	
PCO_2_	>6.0 kpa	4 (5.26%)	49 (30.06%)	1.73×10^−5^
	normal	72 (94.74%)	114 (69.94%)	

Notes: detailed data not recorded in some medical records.

### 5. Comparison between coexisting- defect and non-defect in gastrointestinal tract

We analyzed the relationship between coexisting- defect in gastrointestinal tract and non-defect in gastrointestinal tract, and found that coexisting congenital defects had no significant impact on biochemistry laboratory data(Table 5).

**Table 5:** Comparison of some index between coexisting- defect and non-defect in gastrointestinal tract.

	coexisting defect in gastrointestinal tract(cases)	non-defect in gastrointestinal tract(cases)	P value
Birth weight (kg)	3.19±0.24(14)	3.23±0.45(281)	0.703
length of pyloric muscle (cm)	2.03±0.40(9)	2.08±0.38(259)	0.688
thickness of pyloric muscle (mm)	5.38±1.31(9)	5.38±1.04(259)	0.993
PO_2_ (kpa)	4.22±1.74(13)	4.92±1.51(226)	0.111
PCO_2_ (kpa)	10.13±5.21(13)	9.43±4.29(226)	0.573
HCO_3_^−^ (mmol/L)	29.78±10.47(13)	32.54±10.26(225)	0.349
Serum chloride (mmol/L)	89.81±9.35(10)	88.92±12.91(190)	0.829
Serum potassium (mmol/L)	3.88±1.14(13)	3.74±0.78(226)	0.543
serum sodium (mmol/L)	132.16±3.92(13)	133.65±6.25(228)	0.397
serum pH	7.59±0.12(13)	7.55±0.11(228)	0.229

Notes: detailed data not recorded in some medical records.

## Discussion

IHPS is the most common gastrointestinal disease in the first few weeks of life. The male-to-female ratio is about 4∶1, ranging from 2.5∶1 to 5.5∶1 [1], [2], [3], [4], [16].It is demonstrated that there were 271 boys, male prevalence 271∶45 in our study.

The upper gastrointestinal (UGI) brium series and ultrasound are noninvasive and accurate methods to diagnose infantile hypertrophic pyloric stenosis. The UGI barium series requires oral contrast to indirectly outline the pylorus and the disadvantages are obvious, including exposure to ionizing radiation and the risk of aspiration. Therefore, ultrasound examination has been largely applied to diagnose IHPS. Ultrasonographic examination can be performed with an accuracy approaching 100% in diagnosing IHPS [17], [18]. Many scholars believed the lower limit of abnormal measurement value of pyloric circular muscle thickness of ultrasonograpic examination is within the rang of 3.0–4.5 mm, and with patients presenting between 3.0 and 4.0 mm as “borderline”[14], [18], [19], [20]. A muscle wall thickness <2.0 mm is generally considered normal. According to Hernanz-Schulman [18], the circular muscle thickness ranged from 3.3 to 7.0 mm, and the mean was 4.6±0.7 mm. Our research demonstrate the thickness varied between 3 and 8 mm, the mean was 5.4±1.0 mm, and the diagnostic accuracy by ultrasound was 98.17%.

For the length of the pyloric channel, Ostlie et al [21] found by ultrasound that the mean length of the pyloric channel in infants with pyloric stenosis is 1.95 cm. Many studies [14], [22] found an abnormal length for IHPS ranging from 14 mm to 20 mm; Rohrschneider et al [19] found that pathologic limits were 15 mm for pyloric length. Reed et al [23] believed the abnormal elongation of the pyloric canal is defined as greater than 12 mm in length for IHPS, so many scholars [14], [19], [22] concluded that the muscle thickness was the most discriminating factor and accurate criterion to make the diagnosis, while the length of the pyloric channel is seemingly less important, although this measurement may be useful for the surgeon to perform the pyloromyotomy and in cases in which muscle thickness is within borderline. In our study, the minimum length of pyloric channel was 11 mm, shorter than that reported in the literature. For this infant, the muscle wall thickness was 4 mm, and the diagnosis was confirmed by the upper gastrointestinal (UGI) brium series and operation.

In most cases, the typical projectile, non-bilious vomiting usually occurred between the age of 2 weeks and 8 weeks [2], [13]. Most cases were diagnosed between 3 and 12 weeks after birth [7], and the median age at presentation was 5 weeks (range 0–31 days)[24]. The median age at presentation was 30.8 days (10–77 days)[25]. The median symptom duration between age of symptom onset and age of diagnosis was about 7 to 8 days [24], [26]. Our study demonstrated that the mean onset age was 26.5±26.6 days(range 1–351), and the mean age at presentation was 51.5±38.7 days (range 5–388), the median symptom duration was 25.0±25.2 days (range 1–273), obviously longer than other studies. This indicated that the parents may not have taken it seriously.

For birth weight, Pedersen [27] found 85% of patients had a birth weight between 2500 and 4500 grams, and the mean birth weight was 3310 grams. Wang [9] found more common among infants with birth weights of 2,500–2,999 grams, and found a lower frequency of IHPS among infants weighing 4,500 grams or more. According to Walker [25], there was a mean birth weight of 3238 grams (SD, ±667 grams) in infants with IHPS, while with a mean birth weight of 3538 grams (SD, ±490 grams) in control infants. Our research demonstrated that the mean birth weight was 3.23±0.44 kilograms (range 1.6–4.5 kilograms). The weight at presentation was 3.85±0.89 kilograms (range from 1.75 to 9.00 kilograms).

Though IHPS is characterized by familial occurrence, we found only one pair of monozygotic twins both diagnosed with IHPS, only one affected in another monozygotic twins, and only one affected in both triplets. In addition, there was no report about familiar history in our study.

Other coexisting other congenital defects were found in 65 cases(20.6%). Among them, one other congenital defect was seen in 50 cases, two other congenital defects were seen in 12 cases, three in 3 cases. The cardiovascular system congenital defects were the most common (50.8%, 33/65); followed by the gastrointestinal tract congenital defects (24.6%, 16/65) and central nervous system congenital defects (15.4%, 10/65). The rate of other associated congenital anomalies was higher than that reported by Huang [15].

We further analyzed the relationship between the coexisting congenital defects in gastrointestinal tract and non- coexisting congenital defects in gastrointestinal tract,and found that coexisting congenital defects had no significant impact on biochemistry laboratory data.

The duration of symptoms had an important influence on the biochemical parameters [28], [29]. Oakley [30] found hypochloraemia and alkalosis were independent predictors of IHPS, Gotley [24] found 61% of patients had elevated bicarbonate and 29% of infants had hypochloraemia when admission. While Papadakis [31] reviewed the electrolytes of 283 infants presenting with IHPS and found that 88% had no electrolyte abnormalities on admission. In our cohort, over 84.94%(203/239) of infants had acid-base imbalance, and 58.58%(140/239) had hypoxemia, 74.90%(179/239) had elevated bicarbonate. We found no difference in serun sodium concentration and PH between the two groups. However, there were significant difference between the two groups about the mean Pyloric muscle thickness (5.08±0.95 mm vs 5.53±1.06 mm, p = 0.001), arterial partial pressure of oxygen(10.76±4.27 kpa vs 8.86±4.24 kpa, p = 0.001), arterial partial pressure of carbon dioxide(4.20±1.27 kpa vs 5.20±1.54 kpa, p = 1.62×10^−6^), serun potassium concentration (4.07±0.65 mmol/L vs. 3.60±0.83 mmol/L, p = 2.34×10^−5^), serun chlorine concentration (93.89±11.12 mmol/L vs. 86.69±12.78 mmol/L, p = 0.000158) and bicarbonate level(26.75±6.66 mmol/L vs 34.99±10.59 mmol/L, p = 2.13×10^−9^) (Table 3). The rates of hypokalemia, hypochloraemia and hypercapnia in the early onset group were significantly lower than those of late group (Table 4).

In conclusion, this study shows the onset age of IHPS is 3∼5 weeks in Chinese Han population. The mean pyloric circular muscle thickness is 5.4±1.0 mm, and more than 20% of the patients are accompanied with other congenital defects. Most of the patients suffered hypokalemia, hypochloraemia and hypercapnia. For infants with persistent vomiting at the age of 3∼5 weeks after birth, IHPS should be considered, and go to hospital as soon as possible in order to avoid deterioration.
